# Immune Modulation in Peripheral Blood of Cystic Fibrosis Patients Following Ex Vivo Co-Culture with Mesenchymal Stem Cells

**DOI:** 10.3390/cells15171530

**Published:** 2026-08-25

**Authors:** Halime Mualla Vatansever, Sabriye Senem Kilic, Can Ilgin, Zeynep Tunca, Tunc Akkoc, Emel Eryuksel, Sehnaz Olgun Yildizeli

**Affiliations:** 1 Department of Pulmonology, Marmara University Faculty of Medicine, 34899 Istanbul, Turkey; dreryuksel@gmail.com (E.E.); drsehnazolgun@yahoo.com (S.O.Y.); 2 Department of Immunology, Marmara University Faculty of Medicine, 34854 Istanbul, Turkey; sabriyesenem@gmail.com (S.S.K.); zeyneptunca15@gmail.com (Z.T.); tuncakkoc@gmail.com (T.A.); 3 Department of Radiation Oncology, Istanbul Faculty of Medicine, Istanbul University, 34093 Istanbul, Turkey; can.ilgin@hotmail.com

**Keywords:** immunomodulation, mesenchymal stem cells, peripheral blood mononuclear cells, lymphocyte proliferation, cytokines, dental follicle, regulatory T cells (Tregs)

## Abstract

**Highlights:**

**What are the main findings?**

- In peripheral blood mononuclear cells (PBMCs) obtained from patients with cystic fibrosis, co-culture with mesenchymal stem cells (MSCs) reduced lymphocyte proliferation stimulated by a CD3/CD28 T-cell activating antibody cocktail (CDmix) and increased the proportion of regulatory T cells (Tregs).
- Following MSC co-culture, levels of TNF-α, IL-8, and IL-23 decreased, while significant changes were observed in the levels of IFN-α2, IL-12p70, IL-18, IL-33, and MCP-1, supporting the broad immunomodulatory effects of MSCs.

**What are the implications of the main findings?**

- The findings suggest that MSC co-culture in cystic fibrosis may not only suppress the proinflammatory response, but also induce a multifaceted immunomodulation that rebalances various components of the immune response.
- This study is one of a limited number of studies evaluating the immunological effects of MSC in a peripheral blood-based ex vivo model and lays the groundwork for future mechanistic and clinical research.

**Abstract:**

Background: Cystic fibrosis (CF) is an inherited multisystemic disease. Despite advances in treatment, many patients still experience progressive lung dysfunction. Dental follicle-derived mesenchymal stem cells (DF-MSCs) possess significant immunomodulatory potential in inflammatory airway diseases. We evaluated the effects of DF-MSCs on lymphocyte proliferation, CD4^+^CD25^+^FoxP3^+^ regulatory T cell (Treg) frequency, and cytokine responses in peripheral blood mononuclear cells (PBMCs) from CF patients. Methods: PBMCs from 20 CF patients and 20 matched healthy controls were isolated by density gradient centrifugation, stimulated with a CD3/CD28 T-cell-activating antibody cocktail (CD-mix), and co-cultured ex vivo with cryopreserved DF-MSCs. Lymphocyte proliferation was assessed by carboxyfluorescein succinimidyl ester (CFSE)-based flow cytometry, and Tregs were analyzed by flow cytometry. Cytokine levels in culture supernatants were quantified using a multiplex immunoassay. Results: DF-MSC co-culture significantly suppressed lymphocyte proliferation in CF PBMCs. Treg frequency significantly increased in the stimulated CF samples following MSC co-culture. Following co-culture, levels of tumor necrosis factor-alpha (TNF-α), interleukin-8 (IL-8), and IL-23 decreased. In CF samples, levels of interferon-alpha 2 (IFN-α2), monocyte chemotactic protein-1 (MCP-1), interleukin-12 (IL-12), interleukin-18 (IL-18), and interleukin-33 (IL-33) were elevated. Conclusions: DF-MSCs reduced lymphocyte proliferation, increased Treg frequency, and regulated cytokine levels in CF, supporting their potential to restore immune balance.

## 1. Introduction

Cystic fibrosis (CF) is an autosomal recessive disorder caused by mutations in the Cystic Fibrosis Transmembrane Conductance Regulator (CFTR) gene on chromosome 7 and is the most common genetic disease among Caucasians [1]. Although monogenic, CF shows variable phenotypes, including progressive lung disease, pancreatic insufficiency, diabetes, and growth impairment [2].

CF treatment includes airway clearance, antimicrobial therapies, anti-inflammatory approaches, and CFTR modulators. Although CFTR modulators have improved outcomes in patients with eligible mutations, their benefits are limited to patients with responsive CFTR variants, and they do not fully address the chronic pulmonary manifestations of CF. Consequently, some patients continue to experience progressive lung disease despite advances in treatment [3,4].

Mesenchymal stem cells (MSCs) are multipotent, immunomodulatory, and capable of tissue repair, making them attractive candidates for cell-based therapies [5,6]. They can differentiate into adipocytes, osteoblasts, and chondrocytes and have shown therapeutic potential in several pulmonary diseases, including acute lung injury, chronic obstructive pulmonary disease, idiopathic pulmonary fibrosis, bronchial asthma, and cystic fibrosis, through proposed anti-inflammatory, immunomodulatory, regenerative, and tissue-repair effects [6]. However, in CF specifically, most MSC research remains limited to animal and in vitro airway models [7]. In addition, a small number of early-phase clinical studies have evaluated intravenous MSC infusion in patients with CF, primarily focusing on safety and feasibility. The CEASE-CF Phase I study evaluated intravenous infusion of allogeneic bone marrow-derived MSCs in adults with CF, supporting the feasibility and tolerability of MSC infusion in this population. However, this early-phase study was not designed to establish therapeutic efficacy, and the immunomodulatory mechanisms underlying the potential effects of MSCs in CF remain incompletely understood [8].

Dental follicle-derived MSCs (DF-MSCs) are an accessible and expandable source of MSCs with immunomodulatory properties, making them a promising alternative source for cell-based therapies [9]. In particular, the effects of DF-MSCs on lymphocyte proliferation, regulatory T-cell frequencies, and cytokine responses in peripheral blood samples from patients with CF have not been extensively investigated.

Given the dysregulated inflammatory response in CF and the reported impairment of Tregs, particularly in patients with chronic Pseudomonas infection, Treg-mediated immune regulation may be important for maintaining immune homeostasis [10]. Therefore, this study aims to evaluate the effects of MSC ex vivo co-culture on lymphocyte proliferation, CD4^+^CD25^+^FoxP3^+^ regulatory T cell (Treg) frequency, and cytokine responses in peripheral blood samples from CF patients, contributing to a better understanding of MSC-mediated immune modulation in CF.

## 2. Materials and Methods

### 2.1. Data Collection

Cystic fibrosis (CF) patients aged ≥18 years (*n* = 20), followed at our hospital between October 2022 and November 2023 and without new symptoms or hospitalizations within the past month, were included. Demographic data, pulmonary function tests, current treatments, and bacterial colonization status were recorded, and peripheral blood samples were collected. Age- and sex-matched healthy individuals without chronic disease served as controls. Written informed consent was obtained from all participants.

### 2.2. Peripheral Blood Mononuclear Cell (PBMC) Isolation

Peripheral blood mononuclear cells (PBMCs) were isolated from peripheral blood samples. Briefly, 10 mL of blood was transferred into a 50 mL sterile Falcon tube and diluted with an equal volume of phosphate-buffered saline (PBS). The mixture was carefully layered over Ficoll-Paque and centrifuged at 2000 rpm for 20 min at room temperature. The mononuclear cell layer was collected, washed with PBS, and centrifuged at 1500 rpm for 5 min. The resulting cell pellet was resuspended in complete Roswell Park Memorial Institute-1640 medium (cRPMI; Gibco, Cat. No. 11875-093, Grand Island, NY, USA), stained with trypan blue, and counted using a Thoma chamber (PRECICOLOR HBG, Henneberg-Sander GmbH, Giessen-Lützellinden, Germany; chamber depth: 0.100 mm; grid area: 0.0025 mm^2^).

### 2.3. Dental Follicle-Derived Mesenchymal Stem Cell (DF-MSC) Culture

Previously isolated and cryopreserved DF-MSCs from healthy donors were used in this study. Allogeneic cells were utilized without Human Leukocyte Antigen (HLA) or blood type matching, in line with the literature suggesting that MSCs exert immunomodulatory effects independent of HLA compatibility [11].

After thawing in a 37 °C water bath, the cells were washed with Dulbecco’s PBS (DPBS) containing 2% fetal bovine serum (FBS) and pelleted by centrifugation. Cells were then plated in Dulbecco’s modified Eagle’s medium (DMEM; Gibco, Cat. No. 11885084, Grand Island, NY, USA), supplemented with 10% fetal bovine serum (FBS; Biowest, Cat. No. S1810-500, Nuaillé, France), 100 µg/mL streptomycin, and 100 U/mL penicillin (Biowest), and incubated at 37 °C with 5% CO_2_. At 70–80% confluence (passage 3), cells were washed with DPBS and detached using trypsin-EDTA (Sigma-Aldrich, St. Louis, MO, USA) for subculture.

### 2.4. Surface Marker Characterization of DF-MSCs

At passage 3, DF-MSCs were characterized via flow cytometry. After trypsinization, cells were stained with fluorochrome-conjugated antibodies against MSC markers (CD90, CD73, CD105, CD44) and negative markers (CD34, CD45 and HLA-DR) along with appropriate isotype controls (BD Biosciences, San Diego, CA, USA). These markers were assessed to confirm the MSC phenotype and evaluate potential contamination with hematopoietic and other non-MSC lineages. Analysis was performed on a Beckman Coulter flow cytometer (Marseille, France) using CytoFLEX software (version 2.7).

### 2.5. Differentiation Potential of DF-MSCs

To evaluate the multipotency of DF-MSCs, differentiation into adipogenic, osteogenic, and chondrogenic lineages was induced using StemPro™ Osteogenesis Differentiation Kit (Gibco, Thermo Fisher Scientific, Grand Island, NY, USA; Cat. No. A1007201). Cells (50,000 per well) were seeded onto collagen-coated coverslips in 6-well plates and cultured until 80–90% confluence. Medium was refreshed every three days. After 21–28 days, cells were stained with Oil Red O (adipocytes), Alizarin Red (osteoblasts), and Alcian Blue (chondrocytes) to confirm differentiation.

### 2.6. Peripheral Blood Mononuclear Cell Proliferation Assay

Peripheral blood mononuclear cells (PBMCs) (2 × 10^6^ cells) were labeled with 4 µL CFSE and incubated at 37 °C for 20 min. After staining, cells were washed with RPMI medium and centrifuged at 1500 rpm for 5 min following a 5-min incubation in the dark at room temperature. Anti-CD3 and anti-CD28 antibodies were added to stimulate the cells. Stimulation with anti-CD3 and anti-CD28, which partially mimics the stimulation provided by antigen-presenting cells, provides a signal that can act synergistically with stimulation of the T-cell antigen receptor to activate T-cell proliferation and lymphokine secretion [12,13].

After 72 h of incubation at 37 °C with 5% CO_2_, cells were washed, resuspended in PBS, and analyzed by flow cytometry to assess proliferation. Cell proliferation was assessed using flow cytometry based on CFSE fluorescence dilution; in this method, with each cell division, newly formed cells acquire half the amount of carboxyfluorescein-labeled molecules, and thus each cell division can be assessed by measuring the corresponding decrease in cell fluorescence via flow cytometry [14].

### 2.7. Regulatory T Cell (Treg) Phenotyping

To analyze CD4^+^CD25^+^FoxP3^+^ regulatory T cells, cells were stimulated with anti-CD3 and anti-CD28 antibodies and incubated for 72 h at 37 °C in a 5% CO_2_ atmosphere. Following incubation, cell suspensions were transferred to flow cytometry tubes, diluted with Staining Buffer, and centrifuged at 1600 rpm for 5 min. The supernatant was discarded, and 50 µL of Staining Buffer was added. Surface markers were then added: CD3-FITC (Beckman Coulter, A07746, Marseille, France), CD25-PC5 (Beckman Coulter, IM2646), and CD4-APC (Beckman Coulter, B10874) at 5 µL each. After vortexing, samples were incubated for 20 min at room temperature in the dark.

Then, 3 mL of Staining Buffer was added, and cells were centrifuged at 500× *g* for 5 min. The supernatant was discarded, and the pellet was resuspended in 50 µL of FBS. Subsequently, 2.5 µL of PerFix-nc Reagent 1 was added, vortexed, and incubated in the dark at room temperature for 15 min. After another vortexing step, 150 µL of PerFix-nc Reagent 2 was added. Then, 5 µL of FOXP3-A647 antibody was added, followed by incubation for 1 h at room temperature in the dark. After the addition of 3 mL of 1× PerFix-nc Reagent 3, the samples were centrifuged at 500× *g* for 5 min. The supernatant was discarded, and the pellet was resuspended in 0.5 mL of PerFix-nc Reagent 3. Flow cytometric analysis was then performed.

### 2.8. Co-Culture for Cytokine Analysis

For cytokine measurement, PBMCs were co-cultured with mesenchymal stem cells (MSCs) in 48-well plates at a PBMC:MSC ratio of 6:1. Briefly, MSCs were seeded at a density of MSC cell count: 5 × 10^5^ cells/well in 500 µL of complete medium and allowed to adhere overnight. Next, 3 × 10^6^ PBMCs were added to the corresponding wells. For T-cell stimulation, 0.5 µL of anti-human CD3 and anti-human CD28 antibodies (BioLegend, San Diego, CA, USA), purified using Ultra-LEAF™ (BioLegend, San Diego, CA, USA), was added to each well; no antibodies were added to the unstimulated control wells. The cultures were incubated at 37 °C in a humidified atmosphere containing 5% CO_2_ for 72 h before the supernatant was collected.

### 2.9. Cytokine Measurement

After incubation, 100 µL of supernatant was collected from each well and stored at −20 °C until analysis. Cytokines including IL-1β, IFN-α2, IFN-γ, TNF-α, MCP-1, IL-6, IL-8, IL-10, IL-12p70, IL-17A, IL-18, IL-23, and IL-33 were quantified. A 25 µL aliquot of each sample was analyzed using the Human Inflammation Panel (BioLegend 740809, BioLegend, San Diego, CA, USA) according to the manufacturer’s instructions. Samples were processed with a flow cytometer, and cytokine concentrations were determined using LEGENDplex™ analysis software (v8.0, BioLegend, San Diego, CA, USA) based on standard curves.

### 2.10. Statistical Analysis

Continuous variables were assessed for normality using histograms, Q–Q plots, skewness, kurtosis, and the Kolmogorov–Smirnov test. Data are expressed as medians with interquartile ranges and ranges (min–max). Categorical variables are presented as frequencies and percentages. The Mann–Whitney U test was used for comparisons between independent groups, while the Wilcoxon signed-rank test was used for within-group comparisons. Categorical variables were compared using the chi-square test or Fisher’s exact test, where appropriate. A *p*-value < 0.05 was considered statistically significant. Statistical analyses were performed using Stata version 15.1 (StataCorp LLC, College Station, TX, USA).

## 3. Results

### 3.1. Demographics

A total of 52 adult CF patients were screened, and 20 CF patients (55% female; median age 26 years, range 18–38) and 20 healthy controls (55% female; median age 26 years, range 23–40) were included (Table 1). Bacterial colonization was defined based on the patients’ medical records as persistent isolation of the same bacteria in sputum cultures, rather than a single positive culture. Among the CF patients, sputum cultures showed colonization with *Pseudomonas aeruginosa* in 5 patients (25%), methicillin-susceptible Staphylococcus aureus (MSSA) in 1 patient (5%), both MSSA and *P. aeruginosa* in 10 patients (50%), and no bacterial colonization in 4 patients (20%). Ten out of the twenty CF patients (50%) were receiving elexacaftor/tezacaftor/ivacaftor (ETI) therapy at the time of sample collection. Given the limited sample size, we did not perform subgroup analyses to determine whether age, pulmonary function, or specific comorbidities were associated with the immunological outcomes.

### 3.2. Characterization of Dental Follicle-Derived Mesenchymal Stem Cells (DF-MSCs)

**Figure 1:**
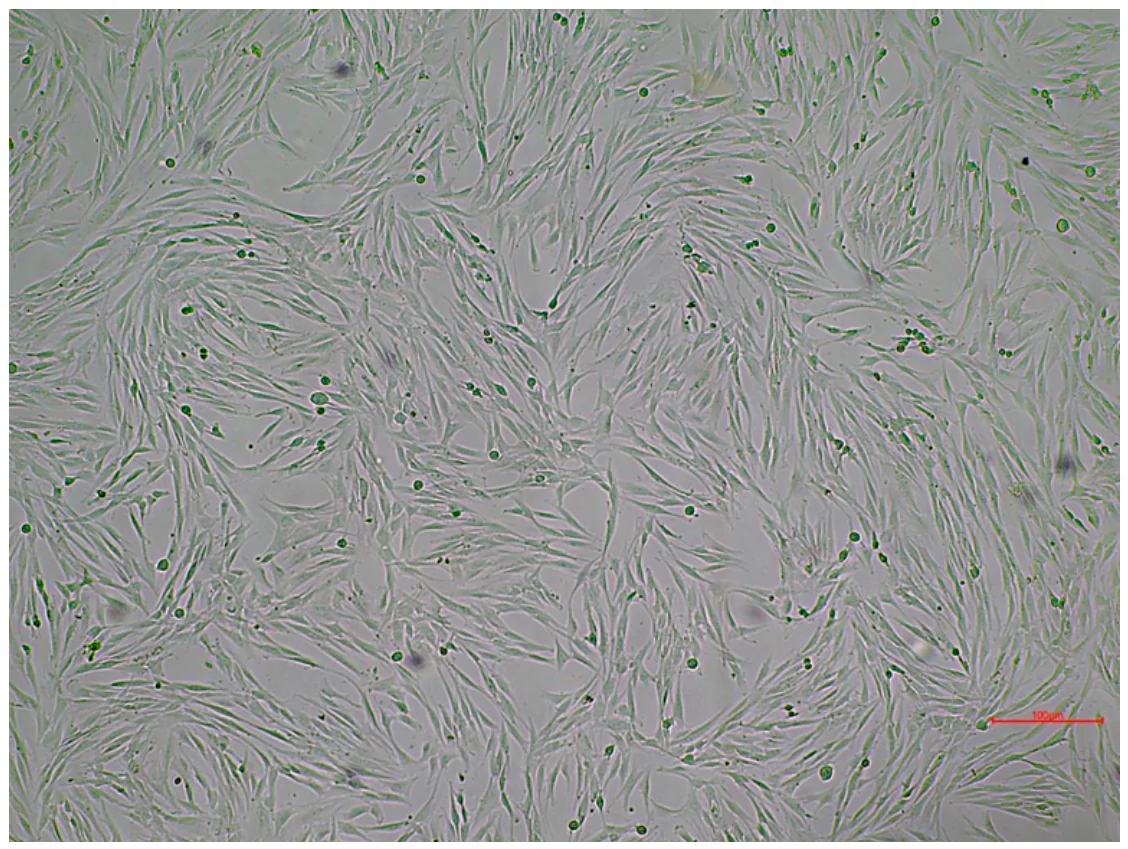
Morphology of dental follicle-derived mesenchymal stem cells (DF-MSCs) at passage 3 (P3). Figure legend: DF-MSCs at passage 3 exhibited a typical fibroblast-like, spindle-shaped morphology under standard culture conditions. The cells adhered to the plastic surface. The image was taken under an inverted microscope. Images were captured at 4× magnification; scale bar = 100 µm.

DF-MSCs were successfully isolated and propagated in vitro. At passage 3 (P3), the cells exhibited a typical fibroblast-like morphology with a spindle shape and homogeneous, adherent growth characteristics under inverted microscopy (Figure 1).

**Figure 2:**
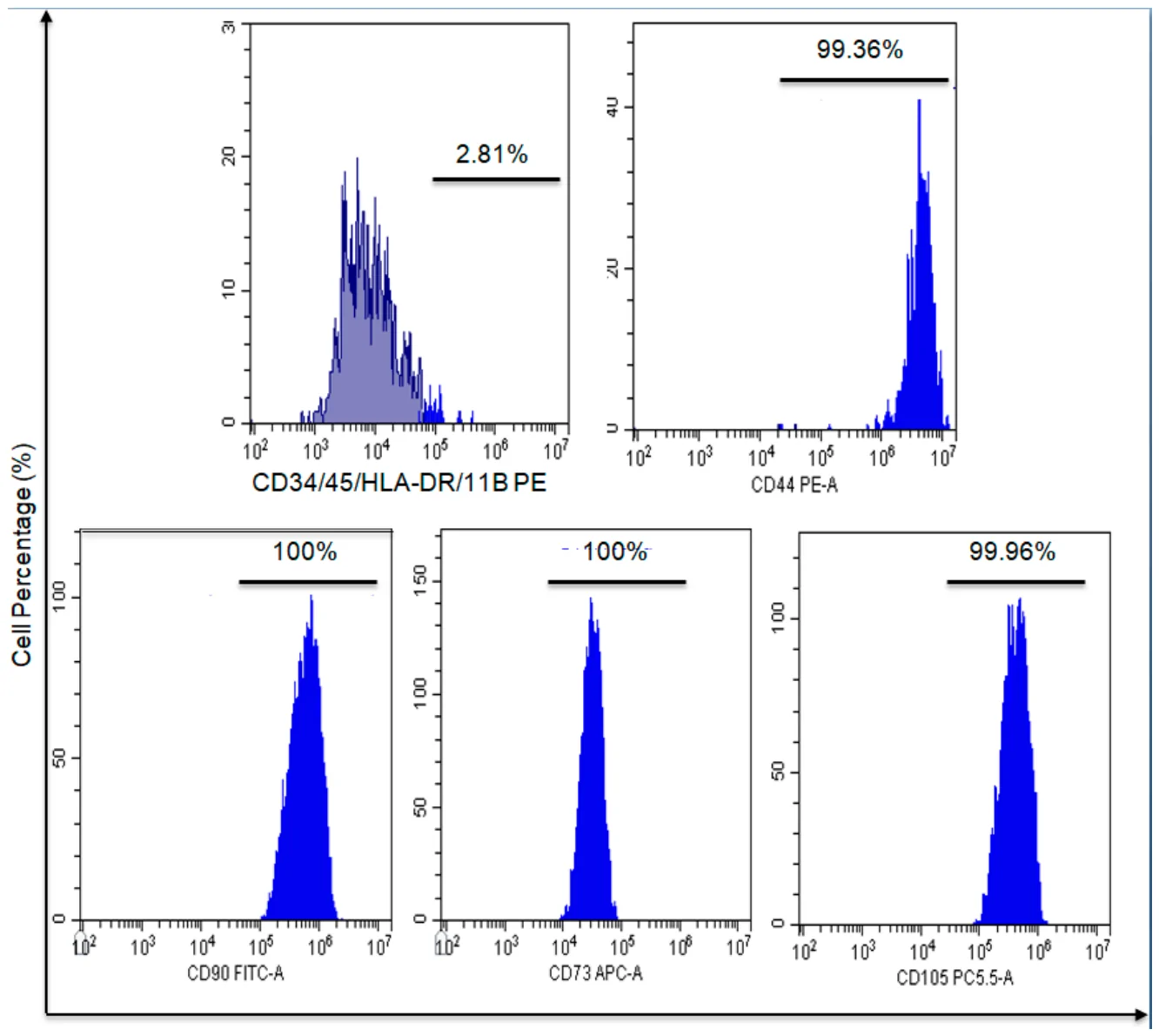
Characterization of dental follicle-derived mesenchymal stem cells (DF-MSCs) by flow cytometry. Figure legend: Flow cytometric analysis of surface markers expressed by dental follicle-derived mesenchymal stem cells (DF-MSCs). Cells were positive for the MSC-specific markers CD90 (100%), CD73 (100%), CD105 (99.96%), and CD44 (99.36%). In contrast, they were negative for hematopoietic and immune cell markers CD34, CD45, and HLA-DR (2.81%). These results confirm the mesenchymal identity of DF-MSCs according to the minimal criteria defined by the International Society for Cellular Therapy (ISCT).

Flow cytometric analysis confirmed the mesenchymal phenotype of the isolated cells. The cells were highly positive for the surface markers CD90 (100%), CD73 (100%), CD105 (99.96%), and CD44 (99.36%) and negative for the hematopoietic and immune markers CD34, CD45, and HLA-DR (2.81%), in accordance with the criteria defined by the International Society for Cellular Therapy (ISCT) (Figure 2). These markers were assessed to confirm the MSC phenotype and evaluate potential contamination with hematopoietic and other non-MSC lineages.

**Figure 3:**
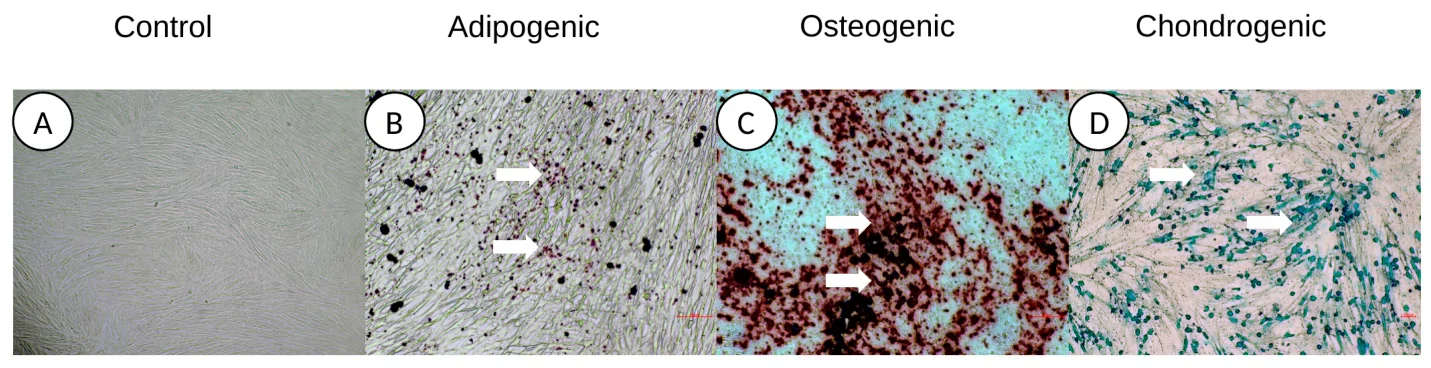
Trilineage differentiation potential of dental follicle-derived mesenchymal stem cells (DF-MSCs). DF-MSCs were cultured under lineage-specific induction conditions to evaluate their multipotent differentiation capacity. (**A**) Control cells maintained in standard growth medium. (**B**) Adipogenic differentiation was confirmed by Oil Red O staining showing intracellular lipid droplets. (**C**) Osteogenic differentiation was demonstrated by Alizarin Red S staining indicating calcium deposition. (**D**) Chondrogenic differentiation was verified by Alcian Blue staining of the glycosaminoglycan-rich extracellular matrix. Images were captured at 4× magnification; scale bar = 100 µm.

To assess multipotency, the cells were induced to differentiate into adipogenic, osteogenic, and chondrogenic lineages. Oil Red O, Alizarin Red S, and Alcian Blue staining confirmed successful differentiation into these lineages. No staining was observed in the control cultures (Figure 3). These results indicate that DF-MSCs retain typical MSC morphology, immunophenotype, and trilineage differentiation potential.

### 3.3. Lymphocyte Proliferation Analysis

The geometric mean of unstimulated proliferation (US) and US after MSC (MSC-US) in CF patients was 5.36 (IQR 0.41–52.83) vs. 17.23 (IQR 2.68–35.27), *p* = 0.12. In the healthy controls, the values were 8.01 (IQR 2.3–14.23) vs. 9.32 (IQR 3.71–43.66), *p* = 0.01. No significant difference was observed between the CF and healthy controls in the changes of US and MSC-US (Table 2A,B).

**Figure 4:**
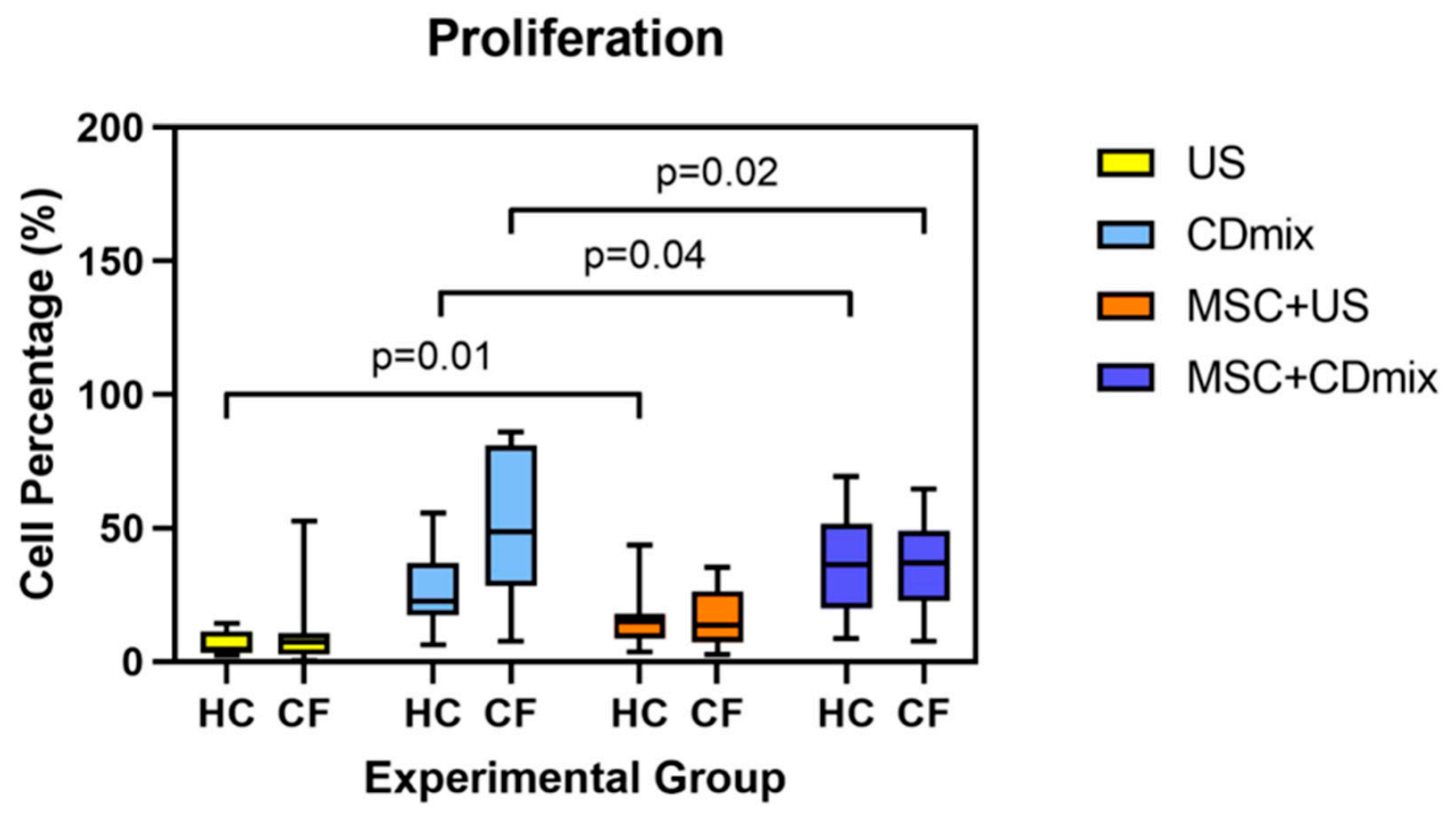
Comparison of proliferation data of cystic fibrosis patients and healthy controls. Figure legend: Proliferation of peripheral blood mononuclear cells (PBMCs) measured by CFSE assay in cystic fibrosis (CF) patients and healthy controls under unstimulated conditions, after anti-CD3/CD28 stimulation (CDmix), and after mesenchymal stem cell co-culture (MSC-CDmix). Data are presented as median and interquartile range (IQR). Statistically significant changes are indicated; detailed statistical results are provided in Table 2A,B.

The geometric mean of stimulated (CDmix) proliferation and CDmix-MSC proliferation in CF patients was 52.36 (IQR 7.57–86.04) vs. 26.45 (IQR 7.55–64.67), *p* = 0.02. In the healthy controls, the values were 19.24 (IQR 6.42–55.93) vs. 29.55 (IQR 8.71–69.46), *p* = 0.04. After ex vivo co-culture with MSCs to the CDmix-stimulated population, proliferation was significantly reduced in the CF group but significantly increased in the healthy controls (Figure 4). A significant difference was detected between the responses of the two groups after CDmix stimulation (*p* = 0.002); however, the response to MSC co-culture in the stimulated group did not differ significantly between the groups (*p* > 0.8) (Table 2A,B).

### 3.4. Lymphocyte Proliferation According to Bacterial Colonization

After MSC co-culture, CDmix-stimulated lymphocyte proliferation significantly decreased in the colonized group, whereas the reduction in the non-colonized group did not reach statistical significance. Unstimulated lymphocyte proliferation increased after MSC co-culture in both groups, although these changes were not statistically significant. Between-group comparisons did not show significant differences in lymphocyte proliferation at baseline or after MSC co-culture. Proliferation data according to bacterial colonization status are presented in Table 3A,B.

### 3.5. Regulatory T Cell (CD4^+^CD25^+^FoxP3^+^) Analysis Following MSC Co-Culture in CF and Healthy Controls

The geometric mean Treg frequency in unstimulated PBMC cultures was 4.12 (IQR 1.28–6.67) at baseline and 1.8 (IQR 0.67–11.63) after MSC co-culture in CF patients (*p* = 0.3). In the controls, the corresponding values were 3.65 (IQR 1.04–9.39) and 3.51 (IQR 1.96–7.35), respectively (*p* = 0.8). The baseline Treg frequency did not differ significantly between CF patients and the controls (*p* = 0.08), and no significant difference was observed after MSC co-culture (*p* = 0.3).

**Figure 5:**
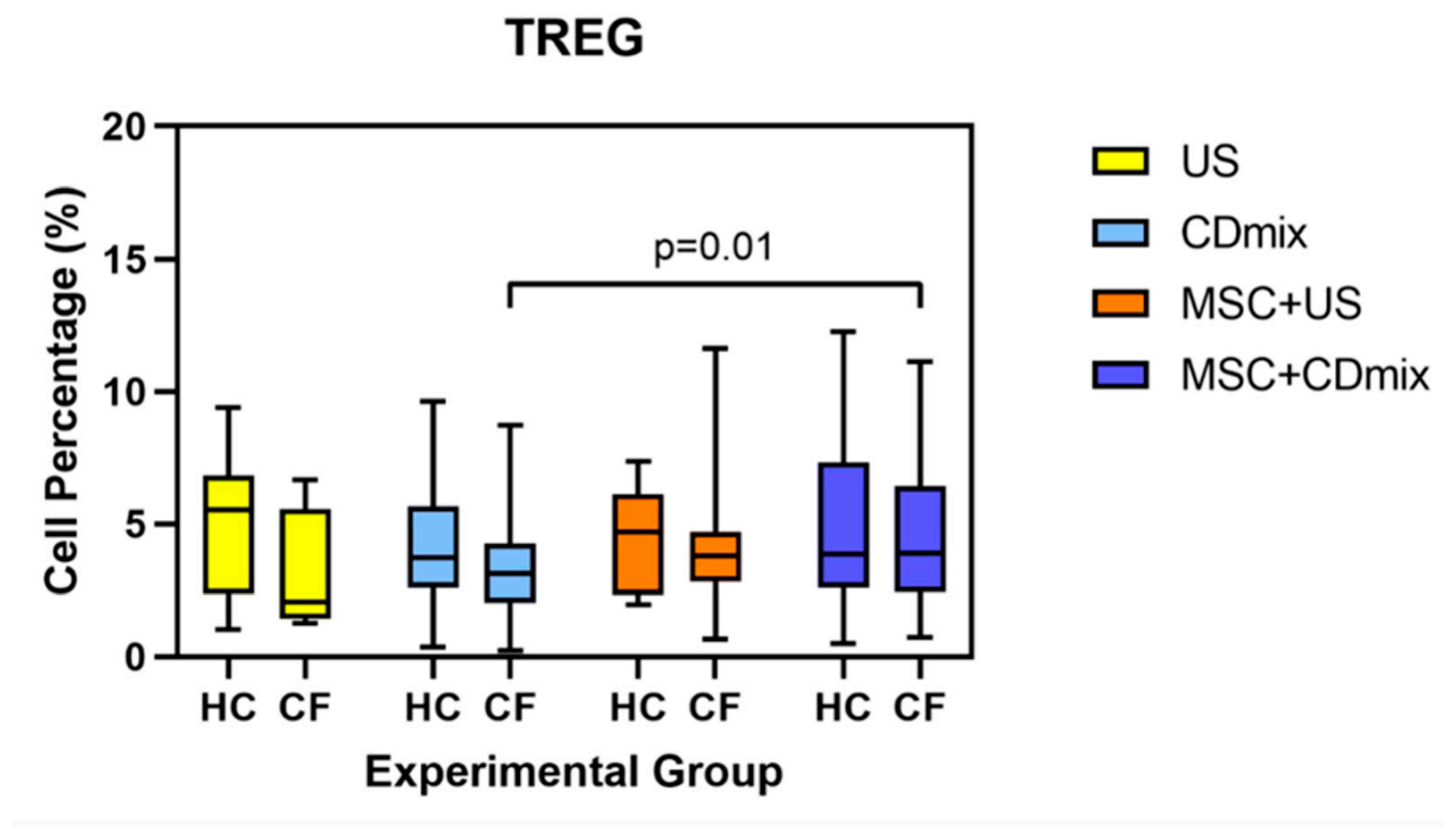
CD4^+^CD25^+^FoxP3^+^ regulatory T cell (Treg) analysis.

For CDmix-stimulated PBMC cultures, the geometric mean Treg frequency was 2.15 (IQR 0.22–8.74) at baseline and increased to 3.7 (IQR 0.73–11.11) after MSC co-culture in the CF patients (*p* = 0.01). In the controls, the corresponding values were 2.9 (IQR 0.35–9.63) and 4.72 (IQR 0.51–12.27), respectively (*p* = 0.5). Thus, MSC co-culture was associated with a significant increase in CDmix Treg frequency in CF patients, whereas the change was not statistically significant in the controls. Despite this within-group increase in CF patients, CDmix Treg frequencies did not differ significantly between the CF patients and controls at baseline (*p* = 0.2) or after MSC co-culture (*p* = 0.6) (Figure 5).

Frequency of CD4^+^CD25^+^FoxP3^+^ regulatory T cells (Tregs) in CF patients and healthy controls under unstimulated conditions, after CDmix stimulation, and after MSC-CDmix. An increase in Treg frequency following MSC-CDmix in CF patients is highlighted.

### 3.6. Cytokine Responses Following MSC Co-Culture

**Figure 6:**
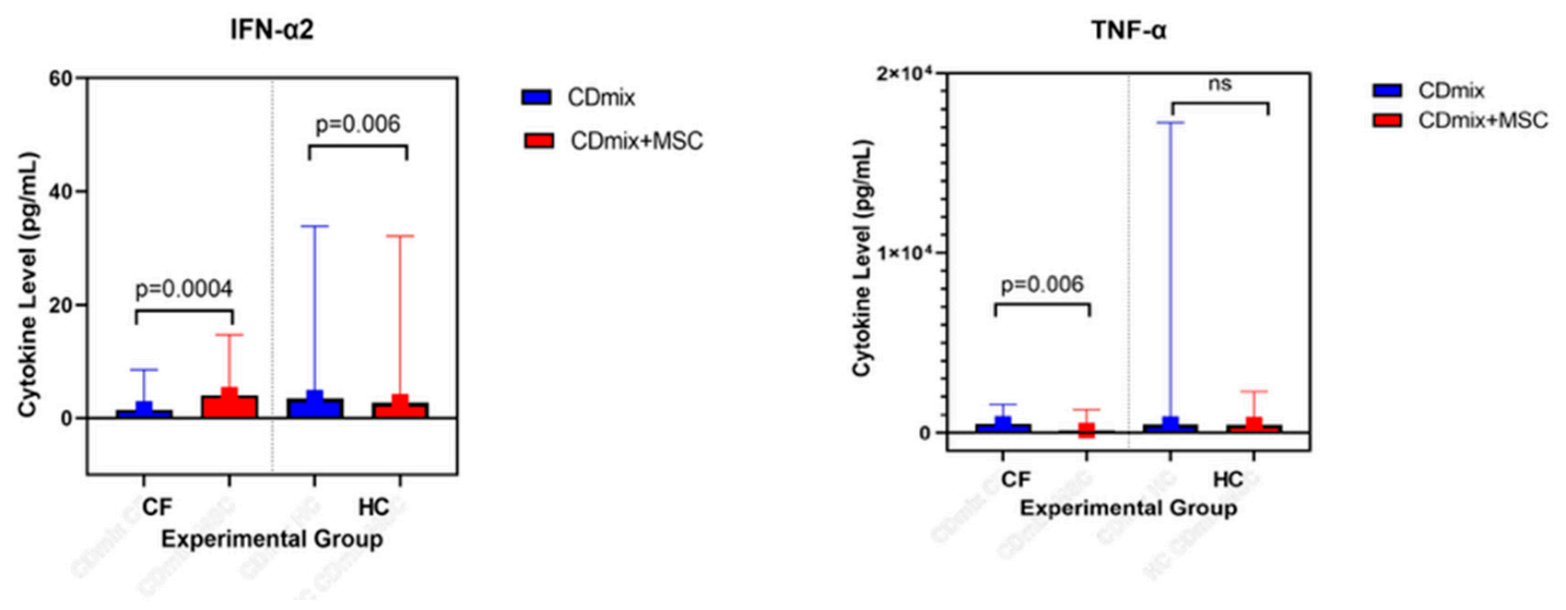

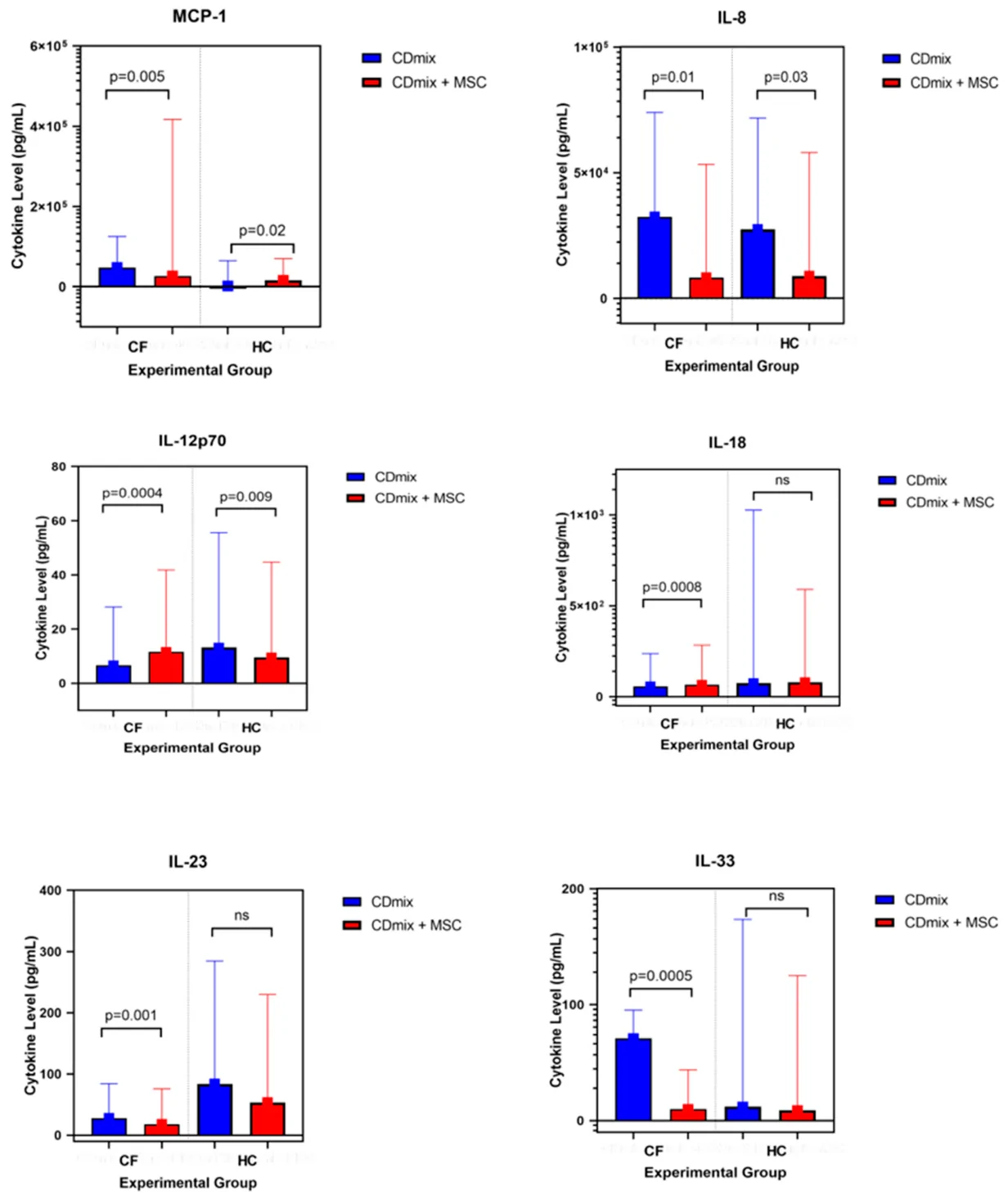
Comparison of cytokine levels in cystic fibrosis patients and healthy controls under CDmix and MSC-CDmix conditions. LEGENDplex analysis of cytokines showing statistically significant changes following MSC co-culture. IL-6 was excluded due to inconsistent results. Data are presented as median and interquartile range (IQR). ns, not significant.

Cytokine levels (IL-1β, IFN-α2, IFN-γ, TNF-α, MCP-1, IL-6, IL-8, IL-10, IL-12p70, IL-17A, IL-18, IL-23, and IL-33) were measured in the supernatant collected from CDmix-stimulated PBMC–MSC co-cultures. Figure 6 presents the cytokines showing statistically significant changes following MSC co-culture.

Full cytokine datasets, including all 12 measured cytokines, are provided in Supplementary Table S1. IL-6 was excluded from the analysis due to inconsistent results.

In CDmix-stimulated PBMC–MSC co-cultures from the CF group, the IFN-α2 levels significantly increased following MSC co-culture (*p* = 0.0004), whereas the IFN-α2 levels significantly decreased following MSC co-culture in the healthy controls (*p* = 0.006). IFN-α2 levels did not differ significantly between the CF and healthy controls before MSC co-culture (*p* = 0.06) or after MSC co-culture (*p* = 0.8).

TNF-α levels significantly decreased following MSC co-culture in the CF group (*p* = 0.006), whereas the reduction in controls was not significant (*p* > 0.05). The magnitude of change between groups was also not significant.

In the CF group, MCP-1 levels significantly decreased following MSC co-culture in CDmix-stimulated PBMC cultures (*p* = 0.005), whereas a significant increase was observed in the healthy controls (*p* = 0.02). Baseline MCP-1 levels in CDmix-stimulated PBMC cultures did not differ significantly between the CF patients and controls, whereas after MSC co-culture, the MCP-1 levels were significantly higher in the CF group than in the healthy controls (*p* = 0.01).

Significant decreases in IL-8 levels were observed in both groups after MSC co-culture (*p* = 0.01 vs. *p* = 0.03), but the magnitude of change did not differ between groups.

The IL-12p70 levels significantly increased after MSC co-culture in CF patients (*p* = 0.0004), while they significantly decreased in the controls (*p* = 0.009). However, these changes did not differ significantly between groups.

MSC co-culture caused a significant increase in the IL-18 levels in CF patients (*p* = 0.0008), whereas the increase in the controls was not significant. The magnitude of these changes also did not differ significantly between groups.

In the CF patients, the IL-23 levels significantly decreased following MSC co-culture in CDmix-stimulated PBMC cultures (*p* = 0.001), whereas no significant effect was observed in the healthy controls. Although the changes in CDmix IL-23 levels did not differ significantly between groups, the IL-23 levels following MSC co-culture were significantly higher in the healthy controls (*p* = 0.01). In the CF patients, the IL-33 levels significantly decreased following MSC co-culture in CDmix-stimulated PBMC cultures (*p* = 0.0005), whereas the decrease observed in the healthy controls was not statistically significant. No significant differences were found between the groups regarding changes in IL-33 levels.

### 3.7. Cytokine Profile in Relation to Bacterial Colonization

Among the CF patients, 5 (25%) were colonized with *Pseudomonas aeruginosa*, 1 (5%) with MSSA, and 10 (50%) with both; 4 patients (20%) had no bacterial colonization. When patients were stratified by colonization status, cytokine findings in the colonized group were consistent with the overall CF results.

Cytokines showing statistically significant differences are presented in Table 4A–C, with the full dataset available in Supplementary Table S2.

## 4. Discussion

In this study, we evaluated the immunomodulatory effects of MSC co-culture on peripheral blood samples from patients with CF and healthy controls. In the CF samples, MSC co-culture significantly reduced CDmix-stimulated lymphocyte proliferation and increased the frequency of CDmix-stimulated Tregs. MSC co-culture also altered the cytokine profile of the CF samples, with differential effects on several inflammatory and immune-regulatory mediators.

Human mesenchymal stem cells (hMSCs) are being investigated in various models of lung disease, such as asthma, interstitial lung fibrosis, and ventilator-associated lung injury [15,16,17]. One of the first preclinical studies to investigate MSC-based therapy in CF was conducted by Bonfield et al. [18] in a murine model of CF lung disease with chronic *Pseudomonas aeruginosa* infection. They reported that hMSC treatment attenuated pulmonary inflammation, reduced pro-inflammatory cytokine levels and lung pathology, and improved bacterial clearance. Their findings suggested that the anti-inflammatory and antimicrobial effects of hMSCs may be mediated, at least in part, by soluble factors such as the antimicrobial peptide LL-37 [18]. Our findings provide complementary evidence of changes in immune responses following MSC co-culture in PBMCs obtained directly from patients with CF. In particular, we observed changes in lymphocyte proliferation, Treg frequency, and cytokine responses following MSC co-culture. Although our ex vivo findings cannot directly establish the mechanisms underlying the effects observed in the murine model, the observed cytokine changes are broadly consistent with the immunoregulatory effects reported by Bonfield et al. [18] and provide complementary evidence from a human CF-derived experimental system [18]. Previous studies have suggested that the immunomodulatory effects of mesenchymal stem cells arise through paracrine mechanisms and interactions with the microenvironment, and that these properties are important in terms of therapeutic potential in clinical applications [8]. Preclinical studies related to mesenchymal stem cells in cystic fibrosis have predominantly used murine models, in which infection and inflammation are induced and immune responses are evaluated via bronchoalveolar lavage (BAL) analyses [18]. Studies examining the inflammatory cascade in cystic fibrosis patients have also identified a possible relationship between airway inflammatory processes and immune markers in the systemic circulation, and therefore, peripheral blood samples may provide a complementary approach for evaluating the inflammatory response [19,20]. Although cystic fibrosis is primarily an airway-centered disease, evidence also suggests that systemic immune dysregulation plays a role in the disease and that there may be a relationship between airway and peripheral immune responses. Furthermore, considering clinical trials (CEASE-CF) in which MSCs have been administered intravenously to cystic fibrosis patients, evaluating the effects of these cells on the systemic immune response is of translational importance [8]. In this context, our study analyzed lymphocyte proliferation and cytokine responses in peripheral blood samples.

In this study, the selection of dental follicle-derived mesenchymal stem cells (DF-MSCs) was based on their accessibility, expandability, and immunomodulatory properties [9]. However, the inherent heterogeneity of DF-MSCs may represent a limitation for their clinical translation, as distinct subpopulations may differ in their proliferative, differentiation, and immunomodulatory properties [21].

In CF, airway inflammation is characterized by a multicellular process involving macrophages and other immune cells, primarily neutrophils, and the chronicity of this response plays a significant role in sustaining persistent inflammation [2]. Bartholomew et al. [22] showed that MSCs suppress lymphocyte proliferation in vitro by using peripheral blood lymphocytes from baboons, including both allogeneic lymphocyte responses and mitogen-stimulated proliferation. The suppressive effect was dose-dependent and could be partially reversed by exogenous IL-2, suggesting that MSC-mediated immunomodulation can directly influence lymphocyte proliferative responses. In their in vivo model, MSC administration also prolonged skin graft survival, further supporting their immunoregulatory properties [22]. However, these findings were obtained in a non-human primate model and not in patients with CF. Our study extends this line of evidence by evaluating lymphocyte proliferation following MSC co-culture using peripheral blood samples obtained directly from patients with CF. In a study by Gornostaeva and Buravkova, it was demonstrated that the inflammatory immune cell microenvironment significantly alters the paracrine profile of MSCs and that MSC responses are influenced by environmental immune activation [23]. In our study, MSC co-culture significantly reduced lymphocyte proliferation in CF samples stimulated with CDmix, whereas an increase in proliferation was observed in healthy controls. When colonized and non-colonized CF patients were evaluated separately, MSC co-culture significantly suppressed proliferation in colonized cases, whereas changes in non-colonized cases did not reach statistical significance. These findings suggest that the altered proliferative response observed in CF may reflect broader CF-associated immune dysregulation rather than a single regulatory mechanism. Although the increase in Treg frequency observed following MSC co-culture may represent one potential mechanism, other factors, including altered effector T-cell responses, cytokine-mediated signaling, and interactions between MSCs and other immune-cell populations, may also contribute. The reduction in proliferation observed in the CF group in our study is consistent with an immunosuppressive effect of MSC co-culture in the context of CF-associated immune activation. In addition, a significant increase in CD4^+^CD25^+^FoxP3^+^ regulatory T-cell (Treg) frequency was observed in PBMC samples from CF patients stimulated with CDmix following ex vivo co-culture with MSCs, whereas no significant change was observed in the healthy controls. Previous studies have reported reduced Treg frequencies in patients with CF, particularly in those with chronic *Pseudomonas aeruginosa* colonization [10]. The increase in Treg frequency observed in our study suggests that MSC-mediated immunomodulation in CF may be associated with changes in the Treg compartment and may contribute to the regulation of the inflammatory response. However, as we assessed Tregs phenotypically by flow cytometry and did not perform a functional suppression assay or determine absolute Treg numbers, these findings do not establish an increase in Treg suppressive activity or absolute Treg expansion. Further studies incorporating functional Treg assays are needed to determine whether the observed change in Treg frequency is accompanied by enhanced suppressive function.

In our study, significant changes were observed in both the proinflammatory and immunoregulatory cytokine axes within the cystic fibrosis immune microenvironment following MSC co-culture. The observed decrease in TNF-α, IL-8, and IL-23 levels is consistent with the suppression of chronic neutrophilic inflammation and the Th17-associated immune response, which are predominant in CF. The decrease in IL-8 suggests the modulation of inflammatory signaling following MSC co-culture. However, as neutrophil chemotaxis and inflammatory cell migration are regulated by multiple chemoattractants and inflammatory mediators, the observed reduction in IL-8 alone cannot establish an effect on neutrophil recruitment or airway neutrophilic inflammation, which plays a critical role in CF pathogenesis. The decrease in TNF-α levels is associated with the suppression of macrophage activation, one of the primary mediators of the systemic inflammatory response; previously, it has been demonstrated that increased TNF-α levels in both BAL and peripheral blood in CF are particularly associated with Pseudomonas colonization [24]. The decrease in IL-23 levels is consistent with the suppression of Th17 differentiation and the IL-17-mediated inflammatory cascade; it is known that the IL-23/IL-17 axis plays a significant role in maintaining chronic airway inflammation in CF [25].

In contrast, the increases observed in IFN-α2, IL-12p70, IL-18, and IL-33 levels suggest that MSCs not only exert an anti-inflammatory effect, but may also mediate a regulatory immune reprogramming process between innate and adaptive immune responses. In this context, while the increase in IFN-α2 and IL-12p70 may be associated with antiviral and Th1-oriented immune activation, changes in alarmin-like cytokines such as IL-18 and IL-33 may reflect a complex response within the MSC–tissue damage–immune activation axis. IL-33 is defined as a key epithelial-derived alarmin playing a role in airway inflammation in CF. Cook and colleagues demonstrated that CFTR deficiency leads to the dysregulation of IL-33 release in the epithelium, and that this effect occurs partially via DUOX1-mediated oxidative signaling pathways. This mechanism leads to the development of increased type 2 inflammatory responses even in the absence of infection. These findings support the concept that IL-33 may act as an upstream regulator of immune activation in airway disease in cystic fibrosis [26].

The observed increase in MCP-1 levels supports the notion that monocyte/macrophage migration and tissue immune cell dynamics may be reshaped under the influence of MSC. When all these changes are considered together, it is believed that MSC co-culture exerts a complex immunomodulatory effect in cystic fibrosis that not only suppresses the proinflammatory response, but also establishes a balance among the various components of the immune system.

The most significant limitation of this study is that subgroup analyses were limited due to the small sample size. The number of patients without bacterial colonization was quite low (*n* = 4). Therefore, subgroup analyses based on bacterial colonization should be considered exploratory and aimed at hypothesis generation rather than confirmatory. Ten patients were receiving ETI therapy; however, subgroup analysis according to CFTR modulator use was not performed because treatment access was inconsistent in some patients, and reliable data regarding treatment duration, adherence, and cumulative exposure were not available. Therefore, the potential impact of ETI therapy on immune parameters could not be evaluated. ETI may have influenced the pre-existing immune state of patient-derived PBMCs and may therefore represent a potential confounding factor when interpreting the effects observed following MSC co-culture. Because the study was not designed to distinguish the effects of ETI from those of MSC co-culture, an interaction between CFTR modulator exposure and MSC-mediated immunomodulation cannot be excluded. Additionally, only peripheral blood samples were used, so no correlations could be established with airway-derived BAL or epithelial cell models. Due to technical limitations, IL-6 analysis could not be included in the study. Since the study was conducted at the in vitro/ex vivo level, it was not possible to assess the clinical relevance of the observed immune changes.

In this study, Treg cells were assessed as a proportion of the analyzed lymphocyte population rather than by absolute cell counts. Therefore, the observed changes in Treg frequency may reflect changes in the relative distribution of Treg cells rather than changes in their absolute numbers. Future studies incorporating absolute Treg counts or Treg-to-effector T-cell ratios may provide a more comprehensive assessment of MSC-mediated changes in Treg populations.

In conclusion, MSC co-culture reduced lymphocyte proliferation in peripheral blood samples from CF patients. Baseline cytokine levels differed between the CF patients and healthy controls. Our findings suggest that MSC co-culture in CF patients may have effects aimed at suppressing inflammation and maintaining immune homeostasis. Further studies are needed to evaluate the clinical implications of these effects.

## Figures and Tables

**Table 1:** Demographics of the patients.

	Cystic Fibrosis (*n*: 20)	Healthy Control (*n*: 20)
Age, year (IQR)	26 (18–38)	26 (23–40)
Gender, women	11 (%55)	11 (%55)
BMI kg/m^2^ (IQR)	20.9 (14.9–28.1)	22.75 (18–31)
Smoking history	0	0
Pulmonary Function Test		
FEV_1_ (Forced expiratory volume in 1 s) (%)	59.5 (IQR 30–127)	91 (IQR 87–110)
FVC (Forced vital capacity) (%)	75.5 (IQR 29–106)	86 (IQR 84–97)
FEV_1_/FVC	83.5 (IQR 82–93)	85.2 (IQR 83–99)
Comorbidities		
Anemia (%)	2 (%10)	0
Diabetes mellitus (%)	7 (%35)	0
Pancreatic insufficiency (%)	19 (95.0)	0
Osteoporosis (%)	4 (%20)	0

**Table 2:** (**A**) Lymphocyte proliferation at baseline and after MSC co-culture in CF patients (*n* = 20). (**B**) Lymphocyte proliferation at baseline and after MSC co-culture in healthy controls (*n* = 20). (**C**) Group comparison (CF vs. Control).

(**A**)			
**Variable**	**Baseline**	**MSC**	***p*** **-Value**
US	5.36 (0.41–52.83)	17.23 (2.68–35.27)	0.12
CD mix	52.36 (7.57–86.04)	26.45 (7.55–64.67)	0.02
(**B**)			
**Variable**	**Baseline**	**MSC**	***p*** **-Value**
US	8.01 (2.30–14.23)	9.32 (3.71–43.66)	0.01
CD mix	19.24 (6.42–55.93)	29.55 (8.71–69.46)	0.04
(**C**)			
**Variable**	***p*** **-Value Baseline**		***p*** **-Value MSC**
US	0.5		0.9
CD mix	0.002		0.8

US data are presented as median (IQR). US: unstimulated; CD mix: anti-CD3+CD28 stimulation; MSC: mesenchymal stem cell co-culture; IQR: interquartile range.

**Table 3:** (**A**) Lymphocyte proliferation at baseline and after MSC co-culture in CF patients with bacterial colonization (*n* = 16). (**B**) Lymphocyte proliferation at baseline and after MSC co-culture in CF patients without bacterial colonization (*n* = 4). (**C**) Group comparison (bacterial colonization + vs. −).

(**A**)			
**Variable**	**Baseline**	**MSC**	***p*** **-Value**
US	5.36 (0.69–52.83)	18.85 (2.68–34.92)	0.2
CD mix	54.65 (7.57–86.04)	26.46 (7.55–53.69)	0.04
(**B**)			
**Variable**	**Baseline**	**MSC**	***p*** **-Value**
US	10.38 (0.41–10.79)	27.56 (7.71–35.27)	0.6
CD mix	47.5 (29.30–83.68)	27.55 (28.75–64.67)	0.2
(**C**)			
**Variable**	***p*** **-Value Baseline**	***p*** **-Value MSC**	
US	>0.05	0.3	
CD mix	0.7	0.1	

Data are presented as median (IQR). US: unstimulated; CD mix: anti-CD3+CD28 stimulation; MSC: mesenchymal stem cell co-culture; IQR: interquartile range.

**Table 4:** (**A**) Cytokine levels in CF patients with bacterial colonization (*n* = 16) before and after MSC co-culture. (**B**) Cytokine levels in CF patients without bacterial colonization (*n* = 4) before and after MSC co-culture. (**C**) Between-group comparisons (colonization + vs. −).

(**A**)			
**Cytokine**	**CD Mix**	**MSC-CD Mix**	***p*** **-Value**
IFN-α2	1.48 (0.54–7.07)	3.07 (2.88–10.76)	0.001
TNF-α	272 (79.03–1085)	157.5 (30.21–1167.67)	0.009
MCP-1	2558 (2–76,979)	18,562 (443–390,507)	0.01
IL-8	32,040 (80–41,620)	8183 (23,582–45,121)	0.02
IL-12p70	5.34 (0.14–21.49)	10.81 (8.60–30.21)	0.01
IL-18	53.33 (0.13–180)	63 (35.10–218)	0.02
IL-23	25.3 (0–56.38)	12.78 (20.44–57.78)	0.03
IL-33	8 (4–24.40)	9.1 (9.80–33.90)	0.01
(**B**)			
**Cytokine**	**CD Mix**	**MSC-CD Mix**	***p*** **-Value**
IFN-α2	1.46 (0.60–2)	7.74 (0.63–8.37)	0.1
TNF-α	863 (16.50–880)	103 (21.02–124.19)	0.6
MCP-1	5240 (261–5502)	10,779 (43,926–54,706)	0.17
IL-8	9811 (2534–35,155)	663 (34,869–35,531)	0.6
IL-12p70	8.43 (0.11–8.54)	25 (0.36–25.41)	0.1
IL-18	64.8 (0–64.82)	160 (2.84–163)	0.1
IL-23	33.33 (2.74–36.07)	57.86 (0–57.86)	0.2
IL-33	7.4 (4.60–12)	22.6 (4.60–27.20)	0.16
(**C**)			
**Cytokine**	***p*** **-Value Before MSC**		***p*** **-Value After MSC**
IFN-α2	0.6		0.3
TNF-α	0.8		0.5
MCP-1	0.7		0.4
IL-8	0.6		0.6
IL-12p70	0.6		0.5
IL-18	0.5		0.2
IL-23	0.9		0.9
IL-33	0.9		0.3

Abbreviations: CD mix = anti-CD3+CD28 stimulation; MSC-CD mix = after MSC co-culture; IQR = interquartile range. Data are presented as median (IQR).

## Data Availability

The data presented in this study are available from the corresponding author upon reasonable request. Due to ethical and privacy restrictions concerning patient-derived biological samples and clinical data, the datasets are not publicly available.

## Supplementary Materials

The following supporting information can be downloaded at: https://www.mdpi.com/article/10.3390/cells15171530/s1, Table S1: Cytokine levels in CF patients and healthy controls before and after MSC co-culture; Table S2: Cytokine levels in CF patients with and without bacterial colonization before and after MSC co-culture.

## Abbreviations

The following abbreviations are used in this manuscript:

APC	Allophycocyanin
BAL	Bronchoalveolar Lavage
CD	Cluster of Differentiation
CD MİX	Anti-CD3 + Anti-CD28 Stimulation Mix
cRPMI	Complete Roswell Park Memorial Institute-1640 Medium
CF	Cystic Fibrosis
CFSE	Carboxyfluorescein Succinimidyl Ester
CFTR	Cystic Fibrosis Transmembrane Conductance Regulator
DF-MSC	Dental Follicle-Derived Mesenchymal Stem Cell
DMEM	Dulbecco’s Modified Eagle Medium
DPBS	Dulbecco’s Phosphate-Buffered Saline
FBS	Fetal Bovine Serum
FITC	Fluorescein Isothiocyanate
FoxP3	Forkhead Box P3
HLA	Human Leukocyte Antigen
IQR	Interquartile Range
IFN-α2	Interferon-Alpha 2
IFN-γ	Interferon-Gamma
IL	Interleukin
MCP-1	Monocyte Chemoattractant Protein-1
MSC	Mesenchymal Stem Cell
MSC-CDmix	MSC-Treated CDmix-Stimulated Condition
MSSA	Meticillin-Susceptable Staphylococcus Aureus
PBMS	Peripheral Blood Mononuclear Cell
PBS	Phosphate-Buffered Saline
RPMI	Roswell Park Memorial Institute Medium
Treg	Regulatory T Cell
TNF-α	Tumor Necrosis Factor-Alpha
