# Supplementary material for: Immune Modulation in Peripheral Blood of Cystic Fibrosis Patients Following Ex Vivo Co-Culture with Mesenchymal Stem Cells
Source: Cells. 2026 Aug 25;15(17):1530. doi: 10.3390/cells15171530 (PMC13564759; doi:10.3390/cells15171530)
Supplement: Supplementary file 1 [file cells-15-01530-s001.zip › cells-4461454-supplementary.pdf]

**Supplementary Table S1: Cytokine levels in CF patients and healthy controls before and after MSC co-culture**

**Table S1A. Cytokine levels in CF patients before and after MSC co-culture (*n*=20)**

| Cytokine | CD mix | MSC-CD mix | *p* (CF) |
|---|---|---|---|
| IFN-α2 | 1.46 (0.54–7.07) | 3.96 (0.63–10.76) | 0.0004 |
| TNF-α | 487.7 (16.54–1085.90) | 124.9 (21.02–1167) | 0.006 |
| MCP-1 | 47966 (2–76979) | 26632 (443.27–390507) | 0.005 |
| IL-8 | 32370 (80.57–41620) | 8183 (23582–45121) | 0.01 |
| IL-12p70 | 6.69 (0.11–21.49) | 11.61 (0.36–30.21) | 0.0004 |
| IL-18 | 56.6 (0–180.16) | 65.27 (2.84–218) | 0.0008 |
| IL-23 | 27.8 (0–56.38) | 17.79 (0–57.80) | 0.001 |
| IL-33 | 71 (4–24.40) | 9.95 (4.60–33.90) | 0.0005 |
| IL-1β | 1097 (29.93-10682) | 1648 (113-13144) | 0.09 |
| IFN γ | 82411 (3.6 – 239575) | 46450 (14.8 – 100843) | 0.2 |
| IL-10 | 908.5 (2.81 – 3698) | 310.3 (48.19 – 1279.7) | 0.4 |
| IL-17A | 209070 (9.8 – 441471) | 178179 (69.9 – 624520) | 0.7 |

**Table S1B. Cytokine levels in healthy controls before and after MSC co-culture (*n*=20)**

| Cytokine | CD mix | MSC-CD mix | *p* (Control) |
|---|---|---|---|
| IFN-α2 | 3.455 (0.67–30.38) | 2.72 (2.02–29.36) | 0.006 |
| TNF-α | 473.3 (21.12–16783) | 449.8 (18.55–1851) | 0.1 |
| MCP-1 | 1721 (7.10–63172) | 15874.9 (288–54082) | 0.02 |
| IL-8 | 27408 (1838.50–44310) | 8829.9 (3211–49216.90) | 0.03 |
| IL-12p70 | 13.2 (0.32–42.40) | 9.6 (8–35.12) | 0.009 |
| IL-18 | 74.4 (7.29–952) | 79.9 (66.70–510.63) | 0.1 |
| IL-23 | 84 (2.17–200.60) | 53.6 (23.04–176.55) | 0.08 |
| IL-33 | 12 (6.60–161.50) | 9 (4–116.10) | 0.2 |
| IL-1β | 222.16 (58.15 – 622800) | 2708 (87 – 10285) | 0.9 |
| IFN γ | 18017 (29.8 – 108816) | 9514 (45.6 – 138267) | 0.8 |
| IL-10 | 1211.55 (2.75 – 84122) | 1069 (85.44 – 45353) | 0.8 |
| IL-17A | 157039 (293 – 517862) | 162250 (1318 – 382144) | 0.6 |

**Table S1C. Between-group comparisons (CF vs Healthy Controls)**

| Cytokine | Δp1 CD mix (CF vs Healthy Controls) | Δp2 MSC-CD mix (CF vs Healthy Controls) |
|---|---|---|
| IFN-α2 | 0.06 | 0.8 |
| TNF-α | 0.4 | 0.2 |
| MCP-1 | 0.9 | 0.01 |
| IL-8 | 0.3 | 0.2 |
| IL-12p70 | 0.07 | 0.09 |
| IL-18 | 0.3 | 0.3 |
| IL-23 | 0.05 | 0.01 |
| IL-33 | 0.2 | 0.2 |
| IL-1β | 0.5 | 0.4 |
| IFN γ | 0.4 | 0.4 |
| IL-10 | 0.8 | 0.3 |
| IL-17A | 0.6 | 0.9 |

Abbreviations: CDmix = Anti-CD3+CD28 stimulation; MSC-CDmix = after MSC co-culture; IQR = interquartile range.

**Supplementary Table S2: Cytokine levels in CF patients with and without bacterial colonization before and after MSC co-culture**

**Table S2A. Cytokine levels in CF patients with bacterial colonization (*n*=16) before and after MSC co-culture**

| Cytokine | CD mix | MSC-CD mix | *p*-value |
|---|---|---|---|
| IFN-α2 | 1.48 (0.54–7.07) | 3.07 (2.88–10.76) | 0.001 |
| TNF-α | 272 (79.03–1085) | 157.5 (30.21–1167.67) | 0.009 |
| MCP-1 | 2558 (2–76979) | 18562 (443–390507) | 0.01 |
| IL-8 | 32040 (80–41620) | 8183 (23582–45121) | 0.02 |
| IL-12p70 | 5.34 (0.14–21.49) | 10.81 (8.60–30.21) | 0.01 |
| IL-18 | 53.33 (0.13–180) | 63 (35.10–218) | 0.02 |
| IL-23 | 25.3 (0–56.38) | 12.78 (20.44–57.78) | 0.03 |
| IL-33 | 8 (4–24.40) | 9.1 (9.80–33.90) | 0.01 |
| IL-1β | 1097 (29.93-10682.11) | 1648.79 (113.27-13144.3) | 0.2 |
| IFN γ | 76773 (7135-152143) | 40589 (14888-62658) | 0.2 |
| IL-10 | 939 (2.81-3698.43) | 315 (48.19-1279) | 0.5 |
| IL-17A | 185853 (9836-441471) | 193590 (69919-624520) | 0.8 |

**Table S2B. Cytokine levels in CF patients without bacterial colonization (*n*=4) before and after MSC co-culture**

| Cytokine | CD mix | MSC-CD mix | *p*-value |
|---|---|---|---|
| IFN-α2 | 1.46 (0.60–2) | 7.74 (0.63–8.37) | 0.1 |
| TNF-α | 863 (16.50–880) | 103 (21.02–124.19) | 0.6 |
| MCP-1 | 5240 (261–5502) | 10779 (43926–54706) | 0.17 |
| IL-8 | 9811 (2534–35155) | 663 (34869–35531) | 0.6 |
| IL-12p70 | 8.43 (0.11–8.54) | 25 (0.36–25.41) | 0.1 |
| IL-18 | 64.8 (0–64.82) | 160 (2.84–163) | 0.1 |
| IL-23 | 33.33 (2.74–36.07) | 57.86 (0–57.86) | 0.2 |
| IL-33 | 7.4 (4.60–12) | 22.6 (4.60–27.20) | 0.16 |
| IL-1β | 745 (188-933) | 800 (406-1207) | 0.17 |
| IFN γ | 239572 (3.64-239575) | 99622 (1220-100843) | >0.05 |
| IL-10 | 1334 (115-1450) | 336 (400-736) | 0.6 |
| IL-17A | 251728 (182-251910) | 163183 (189-163372) | >0.05 |

**Table S2C. Between-group comparisons (colonization + vs -)**

| Cytokine | *p*-value Before MSC | *p*-value After MSC |
|---|---|---|
| IFN-α2 | 0.6 | 0.3 |
| TNF-α | 0.8 | 0.5 |
| MCP-1 | 0.7 | 0.4 |
| IL-8 | 0.6 | 0.6 |
| IL-12p70 | 0.6 | 0.5 |
| IL-18 | 0.5 | 0.2 |
| IL-23 | 0.9 | 0.9 |
| IL-33 | 0.9 | 0.3 |
| IL-1β | >0.05 | >0.05 |
| IFN γ | 0.6 | 0.8 |
| IL-10 | 0.6 | 0.3 |
| IL-17A | 0.9 | 0.9 |

Abbreviations: CD mix = Anti-CD3+CD28 stimulation; MSC-CD mix = after MSC co-culture; IQR = interquartile range.
